# Clinical and Radiological Outcomes of Anterior Approach Microscopic Surgery for the Pincer Mechanism in Cervical Spondylotic Myelopathy

**DOI:** 10.1155/2019/9175234

**Published:** 2019-03-20

**Authors:** Deqing Peng, Yuyuan Ma, Bin Lei

**Affiliations:** Department of Neurosurgery, Zhejiang Provincial People's Hospital, People's Hospital of Hangzhou Medical College, Hangzhou, Zhejiang 310014, China

## Abstract

**Objective:**

We aimed to evaluate the efficacy of anterior approach microscopic surgery for patients with the pincer mechanism in cervical spondylotic myelopathy.

**Methods:**

The clinical data of pincer cervical spondylotic myelopathy that received anterior cervical decompression and fusion in our hospital from Aug 2014 to Dec 2017 were analyzed retrospectively, including 12 males and 9 females, with an average age of 64.3 years (range 46-81 years). Occupying rate, anterior occupying rate, and posterior occupying rate were measured on pre- and postoperative mid-sagittal MRIs. Pre- and postoperative Japanese Orthopedic Association (JOA) scores, intervertebral space height, and C2 to C7 Cobb's angle were analyzed.

**Result:**

Duration of follow-up was six months. The pre- and postoperative anterior occupying rate were averagely 38.6±8.5% and 12.9±5.5%, respectively, the posterior occupying rates were averagely 27.4±7.2% and 13.1±6.6%, respectively, and Cobb's angle changed from 15.3±8.0° to 22.7±7.9°. The intervertebral space height increased from 4.6±0.4mm to 6.5±0.4mm. JOA scores improved significantly by 59.4±34.0% at six months after surgery.

**Conclusion:**

Decompression by anterior microscopic surgery can increase spinal canal volume directly, recover intervertebral space height, and resize Cobb's angle, but decrease the posterior compression by ligament Flava indirectly. Anterior decompression under the microscope may provide an alternative surgical option for partial patients with the pincer mechanism in cervical spondylotic myelopathy.

## 1. Introduction

The cervical spinal cord is compressed not only by the disc protrusions and ossification of the posterior longitudinal ligament (OPLL), but also by the incrassated ligament Flava, which is defined as pincer mechanism [1]. This kind of cervical spondylotic myelopathy (CSM) could be defined as pincer cervical spinal stenosis. The significant spinal cord compression is leading to serious neurological dysfunction, so most doctors agree with surgery to decompression in early stage [2]. The surgical treatment obtains anterior cervical operation and posterior cervical operation in CSM. However, no study could be found in PubMed about microscopic anterior decompression in pincer cervical spinal stenosis.

In this paper, the clinical data of pincer cervical spondylotic myelopathy that received anterior approach surgery in our hospital from Aug 2014 to Dec 2017 were analyzed retrospectively. We compared and analyzed the pre- and postoperative anterior/posterior occupying rate in mid-sagittal MRIs, Japanese Orthopedic Association (JOA) scores, intervertebral space height, and Cobb's angle. An attempt is the first to be made to find out the clinical efficacy of anterior approach microscopic surgery for patients with the pincer mechanism in cervical spondylotic myelopathy.

## 2. Materials and Methods

### 2.1. Patients

Between August 2014 and December 2017, 21 patients diagnosed as pincer cervical spondylotic myelopathy were enrolled, including 12 males and 9 females, with an average age of 64.3 years (range 46-81 years) (Table 1, Table 3). All patients had received anteroposterior, lateral, double oblique, and dynamic lateral over flectional and over extensive cervical spine X-ray, cervical computed tomography (CT) scan, and Magnetic Resonance Imaging (MRI) scan. Cases with continuity ossification of the posterior longitudinal ligament and developmental spinal stenosis were excluded.

X-ray and CT scan could find degenerative cervical spinal stenosis in all patients, manifested as posterior vertebral osteophyte formation and segmental ossification of the posterior longitudinal ligament. MRI scanning showed degenerative bulging disc on the ventral spinal cord and soft tissue compression on the dorsal spinal cord.

All patients suffered from varying degrees of neurological dysfunctions as cervical spondylotic myelopathy (CSM). The onset was typically marked by neck or shoulder complaint, progressive fine motor dysfunction, and decreased hand dexterity, as well as worsening gait and balance. The physical examination showed unilateral or bilateral brachial II triceps, knee, ankle reflex active, unilateral or bilateral positive Hoffman sign and Babinski sign. Upper and lower extremity sensorimotor dysfunction and sphincter disturbance most commonly occur in a slow pattern with disease progression, although rapid neurological decline can occur in a minority of cases.

### 2.2. Surgical Strategy

Neurological dysfunctions and CT/MRI scanning determined the segments of operation. All patients underwent surgery by microscope, which typically takes the form of anterior cervical discectomy and fusion (ACDF). The proper cages were chosen based on adjacent-level standard intervertebral height, which are implanted to recover the intervertebral height and Cobb's angle after fusion. When anteroposterior and lateral plain radiographs were obtained intraoperatively to check correct positioning, anterior plate fixation was inserted.

Drainage was employed for 24-36 hours. Patients can walk on the second day after surgery with the utilization of a cervical collar for nine weeks. The intensive exercise was avoided; antibiotic and hemostasis drug was applied appropriately. The cervical X-ray and MRI scanning were taken after surgery and followed up for six months.

### 2.3. Patient Evaluation and Follow-Up

Lateral radiographs were taken in the neutral position preoperatively and 6-months postoperatively. The intervertebral space height and Cobb's angle were measured for the evaluation of the sagittal alignment of the cervical spine. Cobb's angle is defined as the angle between a line drawn parallel to the inferior endplate of C2 vertebral body and C7 vertebral body at the neutral position. The intervertebral space height of the surgical level was measured as the mean values of the anterior and posterior vertebral body heights. The spinal cord compressed degree was measured by pre- and postoperative anterior/posterior occupying rate in mid-sagittal MRIs (Figure 3(a)). The Japanese Orthopedic Association (JOA) scores (maximum score: 17 points) preoperatively and at 6-months follow-up postoperatively were recorded and compared.

### 2.4. Statistical Analysis

Data analysis was performed with SPSS version 19.0 (SPSS, Inc., Chicago, IL, USA). Student t-test was used to compare preoperative and postoperative anterior occupying rate as well as posterior occupying rate, the intervertebral space height, Cobb's angle, and JOA scores. Statistical significance was set at a level of 0.05.

## 3. Result

All 21 patients underwent ACDF under microscope successfully with a follow-up of 6 months. 7 patients underwent 3-level ACDF, 7 patients underwent 2-level ACDF, and 7 patients underwent 1-level ACDF (Figure 4). Mean operating time was 55 minutes per level (range, 45–70 minutes), and average blood loss was 90 mL per procedure (range, 65–150 mL) (Table 1).

The significant differences were observed in Cobb's angle change from preoperative 15.3±8.0° to 22.7±7.9° of 6-months follow-up. The intervertebral space height increased significantly from preoperative 4.6±0.4mm to 6.5±0.4mm of 6-months follow-up. The pre- and postoperative anterior occupying rate were averagely 38.6±8.5% and 12.9±5.5%, respectively, the posterior occupying rates were averagely 27.4±7.2% and 13.1±6.6%, respectively, and JOA scores improved significantly by 59.4±34.0% at 6th month after surgery (Table 2).

Dural matter tear occurred in resection of ossification of the posterior longitudinal ligament in one patient. Microscopic sutured followed by continuous lumbar subarachnoid drainage for one week after surgery, no cerebrospinal fluid leakage was found. There was no persistent dysphagia, voice complaints, spinal cord injury, tracheal perforations, or oesophageal perforations, wound infection. No cage extrusion or migration occurred.

## 4. Discussion

The spinal cord is recognized with anterior compression of a bony spur and a degenerative bulging disc, and posterior compression of the incrassated ligament Flava (Figure 3(a)), which is defined as the pincer mechanism in cervical spondylotic myelopathy. Taylor [3] first reported this pincer of the cervical spinal cord that compressed between the rear and front occupying lesion with myelography in 1953. Penning presented the pathogenesis as statically and dynamic compression by X-ray and myelography [4]. The static compression factor of the spinal cord arises from the anterior degenerative bulging disc, bony spur, or OPLL, and posterior wrinkled and incrassated ligament Flava. The dynamic compression factor arises from the slip of one vertebral body over the one below, which is defined as dynamic degenerative spondylolisthesis (DS). Sometimes, the slip of vertebral body cannot be found on the standard supine radiographs or MRI. So dynamic lateral over flectional and extensive X-ray is necessary.

It is intuitive that the longer spinal cord compressed, the worse neurological deficit and prognosis would be [2]. It is well established that surgical decompression of the cervical spinal cord is an effective treatment option, not only to stop the progression of symptoms and promote meaningful functional recovery but also to reestablish spinal stability. Surgical decompression can be performed via either an anterior surgical approach or a posterior surgical approach. Anterior surgery typically takes the form of either an anterior cervical discectomy and fusion or corpectomy, and posterior surgery typically involves a laminoplasty or laminectomy and fusion.

The posterior surgery could relieve the posterior compression directly, and the procedure is simple and safe. Sodeyama found that spinal cord moved 2.3mm posteriorly after laminoplasty on average [5]. However, for patients with abnormal cervical lordosis or anterior occupying rate > 50%, the spinal cord posterior movement is limited [5]. Intraoperative damage to the cervical posterior soft tissues including muscles and ligaments causes axial symptoms, and the frequency of such symptoms is reported to be three times following cervical anterior interbody fusion [1, 6]. Authors reported a 10.6-21% incidence of postoperative kyphosis in laminectomy and fusion [7] and 5.3-8.1% incidence of C5 palsy in laminoplasty or laminectomy and fusion [8, 9]. Studies also demonstrated a 13.5-27% reoperation rate after posterior approach surgery [8, 10]. Most surgeons tend to perform posterior surgery first to expand the spinal canal, which could reduce anterior surgery risks in the second stage [11]. Combined anterior and posterior surgery may fully decompress, but presented with a higher incidence of complications, such as excessive bleeding, wound infection, and higher medic cost.

**Figure 1:**
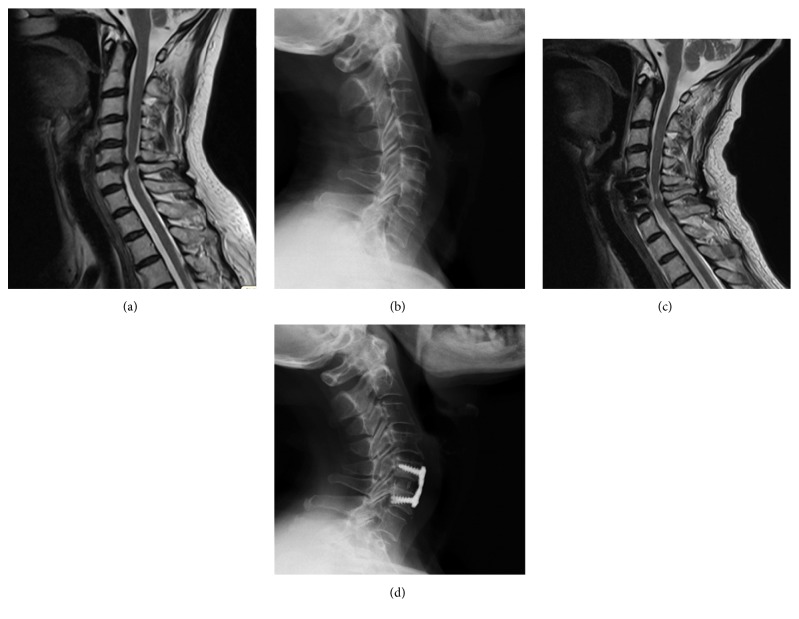
Female, 75-year-old, numbness of right limb, and difficulty in walking for 1 year. Preoperative JOA score was 12. The cervical MR scanning showed C5/6-disc herniation with posterior wrinkled and incrassated ligament Flava, leading to pincer spondylotic myelopathy. Anterior and posterior occupying rates were 36.4% and 36.4% (a). The lateral cervical X-ray showed intervertebral height loss in C5/6. Preoperative Cobb's angle was 9.7° (b). The posterior compression was relieved at the same time by anterior decompression and fusion (ACDF), and the spinal cord was decompressed sufficiently (c). The intervertebral plate and body height were increased after the operation. Postoperative Cobb's angle was 29.1° (d). Postoperative JOA score was 17 at 6th-month follow-up.

Anterior surgery could remove anterior spinal compression such as degenerative bulging disc, bony spur, or OPLL to relieve the static compression directly. All of our patients underwent anterior cervical discectomy and fusion (ACDF) because no multiple level OPLL was included. Fusion with the unstable segment provides stability for eliminating the dynamic compression factor. The loss of height between vertebral bodies narrows the foraminal space and induces secondary pain caused by nerve root compression [12]. Meanwhile, the loss of height gives rise to ligament Flava wrinkle and incrassate. It is crucial to recover intervertebral space height by cage inserted in anterior approach surgery. In accordance with our series and the literature, cage inserted with titanium plate fixation showed a statistically better outcome in Cobb's angle, disc height, and subsidence rate than the stand-alone cage [13]. In our series, Cobb's angle changed from preoperative 15.3±8.0° to postoperative 22.7±7.9° (P<0.05) and the intervertebral space height increased significantly from preoperative 4.6±0.4mm to postoperative 6.5±0.4mm at 6-month follow-up (P<0.05). The anterior occupying rate was reduced from averagely 38.6±8.5% to 12.9±5.5% (P<0.05). Less than 4.3% of ossification of ligament Flava could be found in cervical spine [14]. So, in theory, the recovery of cervical lordosis and disc height could reduce the spinal posterior wrinkled and incrassated ligament Flava, to relieve the posterior compression indirectly (Figure 1). Our series showed the posterior occupying rate changed from averagely preoperative 27.4±7.2% to postoperative 13.1±6.6% (P<0.05). However, the indication for anterior decompression should exclude developmental spinal canal stenosis and severe ossification of the posterior longitudinal ligament.

**Figure 2:**
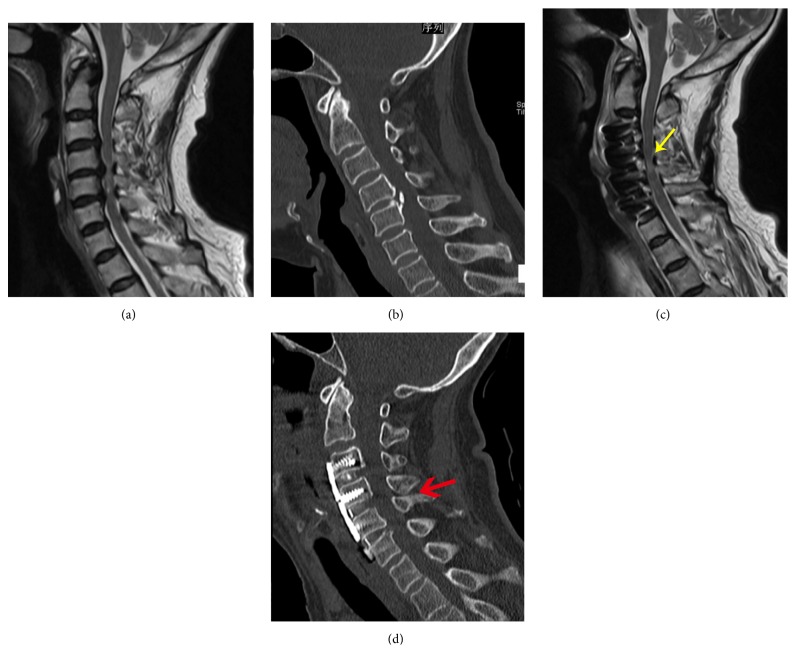
Female, 70-year-old, weakness of limbs for 1 year. Preoperation JOA score was 10. Preoperation Cobb's angle was 10.57° in lateral cervical X-ray. The MR scanning showed C3/4, C4/5, 5/6-disc herniation with posterior wrinkled and incrassated ligament Flava, leading to pincer spinal stenosis. The anterior and posterior occupying rate were 45.4% and 33.3% (a). The sagittal CT imaging showed ossification of the posterior longitudinal ligament (OPLL) in C4/5. (b). The spinal cord was decompressed sufficiently by ACDF of C3/4, C4/5, 5/6 (c). The OPLL was excised by ACDF without corpectomy, while the cage of C4/5 was implanted closer to the anterior vertebral body, leading to intervertebral plate height of C4/5 loss. The wrinkled and incrassated ligament Flava of C4/5 still existed (c&d). Cobb's angle was 28.78°. Postoperation JOA score was 16 at 6th-month follow-up.

**Figure 3:**
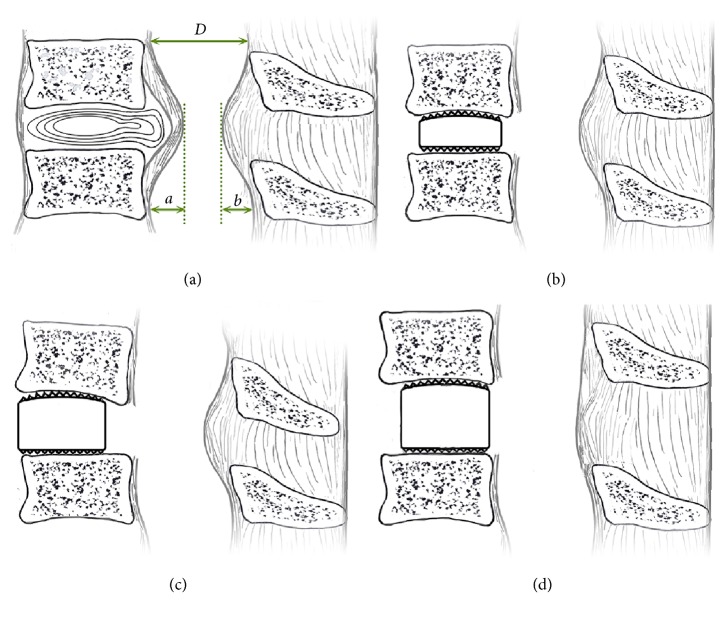
Disc protrusions and ossification of the posterior longitudinal ligament with posterior wrinkled and incrassated ligament Flava, leading to pincer spinal stenosis. Anterior occupying rate = a/D×100%, posterior occupying rate = b/D ×100% (a). The intervertebral body height cannot recover without a suitable cage or cage implanted closer to the anterior vertebral body so that posterior ligament Flava still wrinkled and incrassated (b&c). Suitable cage and suitable implanted position could relieve anterior and posterior compression at the same time (d).

**Figure 4:**
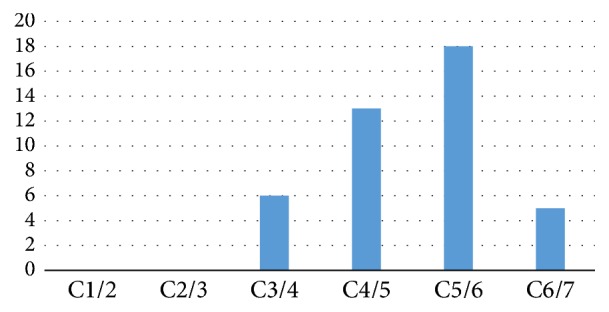
The frequency of surgical fusion levels. The levels are not isolated, and many will be on the same patient.

If the cage was implanted too close to the vertebral body anterior edge, the intervertebral height could not be recovered in vertebral body posterior edge and vertebral plate. The intervertebral space would be wedge-shaped, leading to aggravating the ligament Flava wrinkled (Figures 2(c), 2(d), and 3(c)). So intervertebral retractors should be put deeper to avoid wedge-shaped space and recover disc height completely. In our series, we inserted the cages 2mm from the vertebral body anterior edge. Besides, the intraoperative proper position of the head is essential, and overextension should be avoided while overdistraction of the anterior column may ultimately cause pseudarthrosis, chronic pain, and subsidence [15]. So the intervertebral space should be distracted step by step according to adjacent-level intervertebral space height by intraoperative X-ray. Before the cage was implanted, adequate preparation of both endplates so as not to damage the ossified cartilage is essential.

Because of the limited intervertebral space and illumination deficiency, the surgeon cannot operate with the assistant in the same surgical field. The vertebral body posterior free disc fragments or OPLL always cannot be removed completely due to the risk of injury to pachymeninx, the cord, or nerve root, even the vertebral artery. Traditional anterior surgery may have a high risk of incomplete decompression, limited visual exposure, and injury to the cord. In our series, the operative microscope could provide favorable lighting conditions and amplification for a deep narrow surgical field such as intervertebral space. The compression of the cord and nerve root could be entirely relieved by microsurgical techniques. Besides, bipolar coagulation is easier to stop bleeding from vertebral venous plexus under the microscope. No cord, nerve root, or vertebral artery injury was observed in our patients. Tearing dural mater was sutured under the microscope in one patient, and no cerebrospinal fluid leakage was found. Surgeons may face challenges of microscopic surgery, particularly regarding mastering hand-eye coordination, and also have a learning curve.

The major limitation of this study is the fact that it was not a randomized controlled trial, with a small number of patients, with rather short-term following up. So the result is not established. However, this study may indicate that anterior and posterior decompression could sufficiently achieve together and that the pincer mechanism could be resolved by the anterior approach microscopic surgery.

## 5. Conclusion

This retrospective study indicates that anterior decompression and fusion under microscopic surgery could achieve sufficiently anterior and posterior decompression for patients with the pincer mechanism in cervical spondylotic myelopathy. This minimally invasive technique may have potential advantages and may provide an alternative surgical option. Nevertheless, further large-size studies are required to clarify the efficacy and safety of this anterior approach procedure, to compare with posterior approach decompression surgery.

## Figures and Tables

**Table 1:** Demographics.

Male/female, n	12/9
Median duration of symptoms, mo (range)	8.5 (1–60)
Increased T2 cord signal, n	15
Diabetes, %	15%
Average blood loss, ml (range)	90 (65-150)
Average surgical levels, n	2.0±0.8
Median follow-up, mo (range)	6 (4-12)
JOA recovery rate, %	59.4±34.0%

Abbreviations: Surgical levels, the number of surgically affected levels.

JOA, Japanese Orthopedic Association; full score 17 points.

**Table 2:** Radiographic evaluation.

	Preoperation	Postoperation	P

Cobb's angle	15.3±8.0°	22.7±7.9°	< 0.0001
The height of disc, mm	4.6±0.4	6.5±0.4	< 0.0001
Anterior occupying rate	38.6±8.5%	12.9±5.5%	< 0.0001
Posterior occupying rate	27.4±7.2%	13.1±6.6%	< 0.0001
JOA score	11.0±1.3	15.4±1.5	< 0.0001

Abbreviations: Cobb's angle, C2–C7 Angle.

JOA, Japanese Orthopedic Association; full score 17 points.

P<0.05 is statistically significant.

**Table 3:** Clinical profiles.

Case	Age	Sex	Involved vertebrate	Cobb's angle (°)		Height of disc(mm)		AOR		POR		JOA	
				Pre-	Post	Pre-	Post	Pre-	Post	Pre-	Post	Pre-	Post
1	62	F	6/7	17.6	21.5	5.2	6.5	33.3%	16.7%	20%	8.3%	12	16
2	74	F	4-7	12.5	24.4	4.7	6.5	45.4%	16.7%	27.3%	8.3%	10	14
3	61	F	5/6	24.3	28.5	4.5	6.5	45.4%	16.7%	18.2%	5%	10	15
4	68	M	3-6	29.6	30.7	4.0	6.0	45.4%	16.7%	27.3%	8.3%	9	13
5	68	F	3-5	23.1	33.8	5.0	7.0	50%	30%	25%	20%	9	12
6	77	M	3-6	7.7	9.8	4.0	5.9	54.5%	18.2%	36.4%	27.3%	9	13
7	75	M	5-7	2.9	9.4	4.5	6.2	40%	5%	30%	10%	11	16
8	69	M	4-6	15.2	20.7	4.5	6.0	27.3%	5%	27.3%	13.6%	13	17
9	63	M	4-6	3.1	23.4	5.5	7.0	30%	15%	30%	10%	12	16
10	50	M	5/6	9.6	5.1	5.0	7.0	33.3%	5%	22.2%	11%	12	16
11	46	M	5/6	5.7	13.3	5.0	7.0	33.3%	5%	22.2%	11%	12	17
12	70	F	3-6	10.6	28.8	4.6	6.4	45.4%	15%	33.3%	18.2%	10	16
13	71	M	3-6	14.6	29.9	4.0	6.0	50%	15%	30%	10%	9	14
14	48	M	5-7	15.5	16.6	5.0	7.0	33.3%	5%	22.2%	11%	12	16
15	54	M	3-6	18.9	25.5	4.3	6.0	33.3%	16.7%	15%	8.3%	13	17
16	81	F	4/5	15.1	27.4	4.5	7	30%	10%	40%	25%	11	15
17	49	F	4-6	20.2	22.5	4.5	6.5	44.4%	10%	22.2%	11%	10	14
18	75	F	5/6	9.7	29.1	5.0	7.0	36.4%	10%	36.4%	11%	12	17
19	67	M	4-7	33.8	27.5	4.8	6.5	36.4%	15%	36.4%	18.4%	12	16
20	50	M	5/6	18.4	19.5	4.8	6.0	33.3%	10%	30%	11%	11	16
21	71	F	4-6	12.4	29.6	4.3	6.0	37.5%	15%	25%	10%	12	17
Average				15.3	22.7	4.6	6.5	38.6%	12.9%	27.4%	13.1%	11.0	15.4

Abbreviations: Cobb's angle, C2–C7 Angle.

AOR, anterior occupying rate.

POR, posterior occupying rate.

JOA, Japanese Orthopedic Association; full score 17 points.

## Data Availability

The data used to support the findings of this study are available from the corresponding author upon request.
